# A novel lipid prodrug strategy for sustained delivery of hexadecyloxypropyl 9-[2-(phosphonomethoxy)ethyl]guanine (HDP-PMEG) on unwanted ocular proliferation

**DOI:** 10.1080/10717544.2017.1399303

**Published:** 2017-11-08

**Authors:** Mei Chen, Jiangping Hou, Guilin Tan, Peng Xie, William R. Freeman, James R. Beadle, Karl Y. Hostetler, Lingyun Cheng

**Affiliations:** a Institute of Ocular Pharmacology, School of Ophthalmology and Optometry, Wenzhou Medical University, Wenzhou, Zhejiang, China;; b Department of Ophthalmology, Dazhou Central Hospital, Dazhou, Sichuan, China;; c Department of Ophthalmology, Provincial Hospital Affiliated to Shandong University, Jinan City, Shandong, China;; d Jacobs Retina Center at Shiley Eye Center, Department of Ophthalmology, University of California San Diego, La Jolla, CA, USA;; e Department of Medicine, University of California, San Diego and the San Diego Veterans Medical Research Foundation, La Jolla, CA, USA

**Keywords:** Intravitreal drug delivery, hexadecyloxypropyl 9-[2-(phosphonomethoxy) ethyl]guanine (HDP-PMEG), ocular toxicity, rabbit PVR model, rat CNV model, lipid prodrug, UHR-OCT, retina fluorescein angiography

## Abstract

Proliferative vitreoretinopathy (PVR) is a blinding eye disease and there is no effective pharmacological measure to prevent PVR development. The difficulty comes from lack of potent antiproliferative agent and lack of sustained delivery to cover high-risk time window for PVR to develop. Lipid prodrug of PMEG, hexadecyloxypropyl 9-[(2-phosphonomethoxy)ethyl]guanine (HDP-PMEG), was prepared and was evaluated as a pharmacological adjuvant to surgical management of PVR. A dose-escalation study determined that the highest nontoxic dose for intravitreal use in pigmented rabbits was 3 µg per eye. The genotoxicity of HDP-PMEG was harnessed as a perioperative preventative measure against PVR in a rabbit eye model while the sustained intravitreal pharmacological effect was evaluated on a laser-induced fibrovascular model in rat eye. After intravitreal 3 µg, HDP-PMEG particles in the rabbit vitreous was visible for at least 6 weeks. A single 50-min intravitreal infusion of HDP-PMEG demonstrated significant inhibition of PVR formation when compared with the eyes infused with only BSS (BSS vs. HDP-PMEG: estimate = 1.14, OR = 3.1, *p* = .027). A single intravitreal 104 ng (equivalent to 3 µg for rabbit eye) of HDP-PMEG significantly inhibit laser-induced fibrovascular proliferation in rat eye by 55% (least square mean pixel, BSS = 4763569.5 vs. HDP-PMEG = 2148129.7, *p* < .0001, generalized estimating equation [GEE]). Retinal fluorescein angiography showed the odds for BSS intervened eyes to have higher-rated FA leaking grades were 38.5 times compared with HDP-PMEG treated eyes (*p* < .0001, GEE). Our study results indicate that single intravitreal HDP-PMEG may be a promising ocular drug delivery as a perioperative intervention to prevent PVR reoccurrence following primary surgical management.

## Introduction

Unwanted cell proliferation is a hallmark of many human diseases, including various tumors and cancers. In the arena of visual science, intraocular cellular proliferation is a prominent feature of several ocular disorders, including proliferative vitreoretinopathy (PVR), one of the most common causes of retinal re-detachment (Cardillo et al., 1997; Asaria & Charteris, 2006; Klein et al., 2008; Wang et al., 2009). PVR can arise from various pathologic conditions such as diabetic retinopathy, ocular trauma, and rhegmatogenous retinal detachment, as well as intraocular surgical procedure itself. The current available treatment for PVR is vitreoretinal surgical approach (vitrectomy); however, recurrent membrane formation can lead to retinal re-detachment and permanent impairment of vision (Asaria & Charteris, 2006). Therefore, pharmacological intervention has been considered as an adjuvant to surgical treatment of PVR. Ideally, a therapeutic would be very helpful if it can provide effective drug level at the vitreous-retinal interface for weeks following one-time application after surgical procedure or incident of eye trauma. It has been reported that a median time to develop PVR after an intraocular procedure or ocular trauma is about 2 months (Mietz & Heimann, 1995). Surgically placed intraocular drug release devices have been reported to be effective inhibitors of experimental PVR development (Rahimy et al., 1994; Cardillo et al., 2004) but transient clinical perioperative use of pharmacological intervention has achieved very limited success (Wiedemann et al., 1998; Asaria et al., 2001) or failed to demonstrate the efficacy (Charteris et al., 2004; Wickham et al., 2007). This indicates that inhibition of proliferation requires protracted exposure of tissue to therapeutic levels of the proper therapeutics. A very recent clinical trial using sustained dexamethasone release device (Ozurdex) failed to show clinical efficacy, which highlighted the challenge to control the unwanted cell proliferation at the vitreoretinal interface (Banerjee et al., 2017). Most FDA-approved antiproliferative agents are hydrophilic small molecules and have short half-life after injection into the vitreous (Jarus et al., 1985; Wiedemann et al., 1985; Kwak and D’Amico, 1992). With these therapeutics, either invasive intraocular surgery to implant a slow release device or frequent intravitreal injections are necessary to achieve a prolonged intraocular therapeutic level. With the limitation of the available medical therapies, further exploration of novel pharmacological treatment options is warranted.

Inspired by the crystalline triamcinolone in vitreous for sustained drug release, we reasoned that similar formulation of antiproliferation therapeutics would be very useful for preventative measures against PVR in a format of perioperative application. Several nucleosides have been lipid-derivatized for enhanced cell penetration in antiviral researches (Beadle et al., 2002; Aldern et al., 2003). These lipid prodrugs of nucleosides are minimally water soluble and behave alike triamcinolone when placed into the vitreous of the eye to provide prolonged protection from herpes simplex virus (HSV) infection of the retina (Cheng et al., 2004). By attaching long chain lipophilic alkoxyalkyl groups to the nucleosides, intravitreal therapeutic duration increased by 7 (HDP-P-GCV/GCV) to 20 (HDP-cP-CDV/CDV) fold (Cheng et al., 2002, 2004). We hypothesized that the similar strategy may be a novel and effective way to fight the unwanted cell proliferation at the vitreoretinal interface. PMEG is a strong inhibitor of DNA synthesis and has been evaluated for anti-proliferative activity on anti-tumor treatment (Kramata et al., 1998; Otova et al., 2009). We hypothesized that lipid prodrug of PMEG would be potent and of use as an antiproliferative agent through the vitreous humor delivery. Indeed, a lipid prodrug of PMEG was reported to be potent antineoplastic activity on non-Hodgkin’s lymphoma or myeloma (Reiser et al., 2008; Thamm et al., 2014). We predicted that an alkoxyalkyl prodrug of PMEG should be poorly water-soluble and readily form an intravitreal drug depot like triamcinolone acetonide (TA). Therefore, alkoxyalkyl prodrugs of PMEG might be a valuable local slow release and long lasting antiproliferative and antineoplastic therapeutics for PVR as well as ocular neoplasms such as ocular lymphoma or ocular melanoma.

The current study was undertaken to evaluate a lipid prodrug of PMEG, hexadecyloxypropyl 9-[2-(phosphonomethoxy)ethyl]guanine (HDP-PMEG), for ocular safety and the implication to inhibit unwanted intraocular cell proliferation via intravitreal delivery in a formulation of suspension.

## Materials and methods

### Compound synthesis

**Figure 1.**
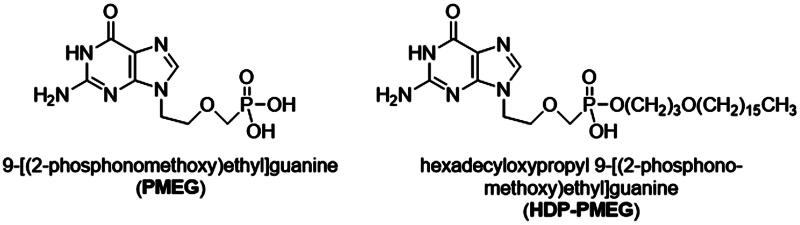
Structures and abbreviations for 9-[(2-phosphonomethoxy)ethyl]guanine (PMEG) and hexadecyloxypropyl 9-[(2-phosphonomethoxy)ethyl]guanine (HDP-PMEG).

The detailed method of preparation of HDP-PMEG was reported previously (Valiaeva et al., 2006). In brief, the diisopropyl ester was converted to the free phosphonic acid, and then esterified with 3-hexadecyloxy-1-propanol (HDP) using the coupling reagent 1,3-dicyclohexylcarbodiimide. To obtain the HDP ester of PMEG, the ester of PME-2-amino-6-chloropurine was prepared and hydrolyzed to PMEG ester by heating in 1 N HCl/ethanol. Most recently, we also reported an alternative synthesis method of alkoxyalkyl PMEG analogs (Beadle et al., 2016). The synthesized crystalline prodrug of PMEG bears a 16-carbon chain tail (Figure 1) and is minimally water soluble, which fits the design for intravitreal delivery to achieve slow-release and long-lasting.

### *In vivo* dose escalation design

A total of 48 adult Chinchilla pigmented rabbits (mean body weight 2.1 ± 0.17 kg) were used for this safety study in accordance with ARVO Statement for the use of Animals in Ophthalmic and Vision Research. Only one eye of each rabbit was used for HDP-PMEG intravitreal injection and the fellow eye was injected with the equal volume of 5% dextrose and used as an internal control. Intravitreal injection was performed under direct view of a surgical microscope using a 1 cc syringe attached with a half inch 27 gauge needle as described previously by us (Cheng et al., 1999). In this study, the injection volume was 50 µl and paracentesis was not performed. Four eyes of 4 animals were used to evaluate each dose of HDP-PMEG at two study end points: 4-week (24 rabbits) and 8-week (24 rabbits). From our in vitro cytotoxicity study, HDP-PMEG was roughly 10 times more cytotoxic than 5FU on Müller cells that are the backbone of retina normal structure. A study reported that the repeated 500 µg intravitreal injection of rabbit eye was the highest nontoxic dose for 5FU (Stern et al., 1983). If we use 5FU as a reference and use 10 times less dose, 50 µg of HDP-PMEG per eye would be the dose to start. Altogether 6 doses were tested using balanced salt solution (BSS) as a diluent: low doses (L) of 1 µg/eye and 3 µg/eye, middle doses (M) of 8.7 µg/eye and 25.7 µg/eye, and high doses (H) of 44.2 µg/eye (50 µg was the targeted dose) and 76 µg/eye. These doses presented a log scale of escalating of 0, 0.47, 0.94, 1.41, 1.645, and 1.88 that corresponded to 76 µg/eye. Those doses were confirmed with the DU 800 Spectrophotometer (BeckMan DU800 Nucleic Acid/Protein Analyzer).

### Clinical ocular safety and electrophysiology of the retina

After the intravitreal injection, both eyes of each animal were monitored by slit-lamp biomicroscopy, indirect ophthalmoscopy, and tonometry (Mentor Tono-Pen XL, Medtronic Solan) on post-injection week 1, week 2, week4, week 6, and week 8. The anterior segment congestion, anterior chamber migrating cell count (Krzystolik et al., 2002), lens clarity and vitreous clarity, and fundus evaluation were performed as we previously described (Cheng et al., 1999). Anterior segment photograph or fundus photograph was taken as necessary. Electroretinographies (ERGs) were performed on post-injection week 1, week 4, and week 8. For ERG examination, the procedure was similar to the previously described methods (Cheng et al., 2004). All stimuli were presented in a Ganzfeld dome (Roland Q400, Wiesbaden, Germany). Dark-adapted ERG was elicited with the flash light intensity of 0.02 cd•S/m^2^, 0.2 cd•S/m^2^, and 2 cd•S/m^2^. Five flashes were averaged. After dark adapted ERG, light adapted for 2 min under the light intensity of 25 cd/m^2^ and single flash with five-flash average photopic ERGs with the flash light intensity of 2 cd•S/m^2^ were recorded. Finally, 20 Hz flicker ERGs were recorded with the same light intensity as the photopic ERGs against the background light of 25 cd/m^2^.

### Histology of the retina

All animals were sacrificed on post-injection week 8 and histology was evaluated with light microscopy (Cheng et al., 1999). For histology study, the globes were dissected vertically through the optic nerve head to ensure the inferior retina to be evaluated because a localized retinal toxicity might be present from drug crystal in contact with inferior retina although ERG could be normal (Cheng et al., 1999, 2004). The rabbit weight was recorded at the beginning and the end the study.

### Genotoxicity of HDP-PMEG for PVR management

Drug genotoxicity has been harnessed for management of the eye diseases, such as mitomycin C on anti-glaucoma filtering surgery (De Fendi et al., 2013) or 5-fluorouracil added into infusion solution during TPPV (Trans Pars Plana Vitrectomy) for prevention of PVR (Asaria et al., 2001). The genotoxicity of PMEG has been long recognized (Otova et al., 1997). For the genotoxicity study, total 18 adult Chinchilla pigmented rabbits were used, 9 used for control and 9 used for genotoxicity evaluation. The rabbits were randomized into the two groups by body weights stratified by the gender using the SAS Proc Plan program. Only one eye of the each rabbit was used for the study procedure while the contralateral eye was untouched. All the animal handlings and the procedures were in accordance with ARVO Statement for the use of Animals in Ophthalmic and Vision Research. Before entering the study, all the rabbits were subjected to routine ophthalmic exams to confirm the eye normality. Under the general anesthesia by intramuscular injections of ketamine (35 mg/kg) and Xylazine hydrochloride (5 mg/kg), 0.4 mL of C3F8 gas was intravitreally injected to compress and liquefy the vitreous of the right eye to facilitate the subsequent cell injection. Seventy-two hours later, fluid-gas exchange was performed and the vitreous cavity infusion of HDP-PMEG at 3 µg/mL for 50 min while the control rabbit eyes were infused with BSS. Subsequently, 4.5 mL of BSS was used to flush the vitreous cavity for 2 min to wash away the HDP-PMEG before injection of primary homologous rabbit retinal pigment epithelium (RPE) at a dose of 200,000 in 0.1 cc per eye. These primary RPE cells have been reported to induce PVR previously (Hou et al., 2015). After the intravitreal injection of RPE cells, the rabbit eyes were followed up on day 4, day 7, day 14, day 21, and day 28 to grade the PVR severity using an indirect ophthalmoscope by a masked examiner according to the published 0 to 7 scales reported by Hida et al. (Hida et al., 1987; Agrawal et al., 2007).

### Intravitreal sustained drug efficacy on laser-induced rat CNV model

Intravitreal injection of rabbit primary homologous RPE cells has been used to evaluate investigative new therapeutics for PVR. However, variations can come from many sources, which can adversely affect the scientific comparison among the studies. For example, cell viability (viability itself pertinent to that batch of cultured RPE cells or viability change from time difference between preparation to the injection) or the status of the vitreous liquefaction (directly affect the distribution of the injected RPE cells) can be very influential to the PVR severity (Agrawal et al., 2007). These variables are difficult to control to be uniform from the study to study. Laser-induced rodent choroidal neovascularization (CNV) has been used as an in vivo economic model of wet form of age-related macular degeneration (AMD) (Koh et al., 2004; Cheng et al., 2010). This model does not involve exogenous cell components and laser power or density can be well controlled via the parameters selection. More importantly, this pathology possesses the both local inflammation and VEGF overexpression, which are the driving forces behind many retinal diseases including PVR. With the improved rodent fundus imaging technology, VEGF overexpression and associated fluorescein leakage can be objectively quantitated by retinal fluorescein angiography (FA). In addition, the laser-induced fibrous vascular proliferation can be quantitated by the advanced optical coherence topography (OCT) technology (Sulaiman et al., 2015).

For the current study, 12 Brown Norway rats were used in accordance with ARVO Statement for the use of Animals in Ophthalmic and Vision Research. The both eyes of each rat were subjected to laser but only right eyes received intravitreal injection of HDP-PMEG while the contralateral eyes received intravitreal BSS as internal control. For the retinal laser coagulation, 810 nm diode laser was used with exposure time of 100 mS, power of 240–280 mW, and spot size of 75 µm. Four laser burns were placed around the optic disc. The laser burns were delivered through a rat cornea contact lens (rat ocular fundus lens, OFA5.4; Ocular Instruments, Inc., Bellevue, WA) and the burns were documented according to the locations as superior (S), nasal (N), inferior (I), and temporal (T) for the both eyes. Only the laser burns with breach of the Bruch’s membrane (air bubble formation) were documented as effective burns. In addition, the bleeding from the laser burns was also documented and graded as 0 if no bleeding, as 1 if the bleeding confined within the laser burn, and as 2 if the bleeding extended beyond the laser burn. For the intravitreal injection, 6 µL of the volume was used, either with BSS or 104 ng of HDP-PMEG. The HDP-PMEG dosing was based on the nontoxic dose of HDP-PMEG in rabbit eye, which was 3 µg/eye. Rabbit eye has a vitreous volume of 1500 µL while the vitreous of an adult rat eye is about 52-56 µL (Berkowitz et al., 1998; Sha & Kwong, 2006–07). The injection was delivered through a 30-gauge needle attached to a 6 inches extension tubing connected to a 250 µL Hamilton syringe on a dispenser as we previously described (Cheng et al., 2010; Chen et al., 2015).

After the laser and interventional intravitreal injection, the rats were examined on day 14 using Phoenix Research Labs Micron IV retinal imaging system to record retinal FA. The images from both the eyes were acquired at the similar times in pairs at early (within 1 min), mid (around 3 min), and late phase (6 min) of FA (see supplemental figure 1). The fluorescein leakages from the effective burns were graded according to our previously published criteria: 0 (no leaking), 1 (minimal intensity increase), 2 (moderate intensity increase), and 3 (both intensity and area increase) (Cheng et al., 2010).

On day 24 after the laser, the OCT imaging was performed with Ultra-High Resolution Optical Coherence Tomography (UHR-OCT) (Zhu et al., 2011; Jiang et al., 2012; Gu et al., 2015). UHR-OCT uses a superluminescent diode (SLD; T840; SuperLum Diodes Ltd., Moscow, Russia) and was adapted onto a slit-lamp system with the installation of an 90 diopter ocular lens (Volk Optical, Inc., Mentor, OH, USA) on the sample arm for rat eye imaging (Zhu et al., 2011; Jiang et al., 2012). The UHR-OCT was configured as follows: scan speed 12000 scans per second, center wavelength 840 nm, bandwidth 100 nm, scan width 18 mm, image size of each B-scan contains 1365 pixel by 2048 pixel. The axial resolution is 3 µm. Each eye had three sets of scans along each axis. For the horizontal section, the scanning laser moved parallel to the X-axis; the scanning started from inferior and move towards superior eye globe. For the vertical section, the scanning laser moved parallel to the Y-axis; the scanning started from right side of the globe towards the left side of the globe. Each set contains 32 cross sections and total of 192 (((32 × 3) × 2)) cross sections were acquired from each eye globe. All sections were reviewed and the laser burns identified on the OCT sections with the guide of color fundus photos (see supplemental figure 2). The greatest linear dimension (GLD) of the base and height of the laser lesions on the OCT sections were used to derive (average) the radius (a, b, and c) of the ellipsoidal laser lesion as described by Sulaiman et al. (2015). The lesions were well identified from the RPE level and above; therefore, the base of the lesion was measured at the bottom of the RPE band on OCT and the height was measured from the apex of the lesion in retina to the base of the lesion (see supplemental figure 2). The volume of the ellipsoidal lesion was calculated using the formula of *V* = 4/3πabc (Sulaiman et al., 2015). The a is the ½ of the lesion base measured on the horizontal scan and the b is the ½ of the base measured on the vertical scan while c is the average of the lesion height from both horizontal and vertical scans.

To confirm the dose safety in rat eye, ERGs (dark, light, and flicker) were acquired from the both eyes before the sacrifice on day 28 following the laser. Upon the sacrifice, the eye globes were enucleated and fixed in the Davidson fixative for 24 h before routine processing for histology and light microscopy.

### Data statistical analysis

For the continuous data such as intraocular pressure (IOP) and ERG amplitude (acquired multiple times from a same animal), or the fibrous tissue volume of the laser burns (multiple burns in the same eye of the same animal), were analyzed and compared among the groups using generalized estimating equations (GEEs) while adjusting for the intra cluster correlations within the same animal (SAS version 9.4, Cary, NC 27513-2414). For the grades of PVR or levels of fluorescein leakage from the laser burns during FA were ordinary data and were sampled multiple times from a same rabbit eye or multiple laser burns from the same eye of a rat. Therefore, GEE ordinal model for multinomial data was applied to analyze the log odds ratios comparing study and control groups to adjust for inter animal or inter eye dependence.

## Results

### Intraocular safety and toxicity

After intravitreal injection of various doses of HDP-PMEG, the findings by slit-lamp biomicroscopy and indirect ophthalmoscopy at week 2 (short term) and week 8 (long term) were summarized in Table 1.

**Table 1.** Clinical observation.

			2 Weeks					8 Weeks	
Dose group	Number of eyes	Anteriorchamber cell count*	Lens clarity	HDP-PMEG visible**	Localized retina WTN/HM#	Anterior chamber cell count	Lens clarity	HDP-PMEG visible	Localized retina atrophy
76.5 µg	4	¾(2)	0	4/4(+++)	0	0	0	4/4(+)	0
43.9 µg	4	2/4(1.5)	0	4/4(++)	2/4	2/4(1)	0	4/4(+)	2/4
27.6 µg	4	2/4(1)	0	4/4(+)	0	0	0	4/4(+)	0
8.7 µg	4	2/4(1)	0	4/4(+)	0	0	0	4/4(+)	0
3 µg	4	0/4(0)	0	4/4(+)	0	0	0	0	0
1 µg	4	0	0	0	0	0	0	0	0

* : Number of eyes showing cells/number of eyes examined (average score of AC cells grading).

** : “+” = Diffused drug particles seen in vitreous; “++” = aggregated drug filament in vitreous; “+++” = aggregated drug patch in vitreous.

# : localized retina whitening and/or hemorrhage.

**Figure 2.**
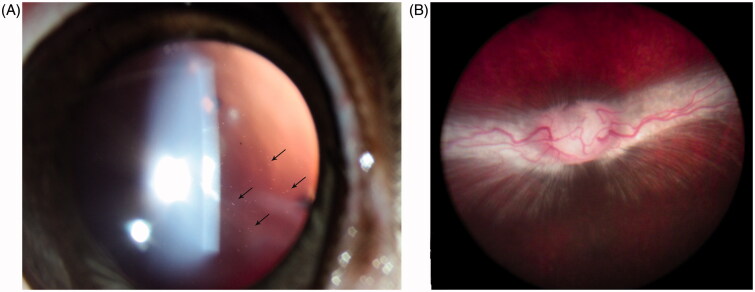
A. Slit-lamp photograph taken 6 weeks after 3 µg HDP-PMEG intravitreal injection, showing fine particles in vitreous (arrows) using retro illumination. B. The fundus from the same animal at the end of this study (8 weeks), showing normal optic nerve head, normal retina, and normal medullary ray.

Three micrograms of HDP-PMEG was determined as the highest nontoxic dose with visible drug particles (drug depot) in vitreous for at least 6 weeks (Figure 2A) and normal fundus at the end of this study (Figure 2B). The higher doses caused various inflammatory reactions in the anterior chamber and retinal hemorrhages or areas of retinal whitening (Table 1).

IOP from the different checking points were pooled to increase the data stability and was analyzed using GEE. The analysis revealed that IOP was not significantly different among the checking points but IOP was significantly different among the dose groups (*p* = .0029). The further analysis of the least square means of the groups showed that IOP of the eyes with higher doses (76 µg (9 mmHg), 44.2 µg (7.8 mmHg), 25.7 µg (9.9 mmHg)) was significantly lower than that of the eyes with lower doses (1 µg (18.8 mmHg), 3 µg (15.3 mmHg), 8.7 µg (14 mmHg)), or control (14.4 mmHg).

**Figure 3.**
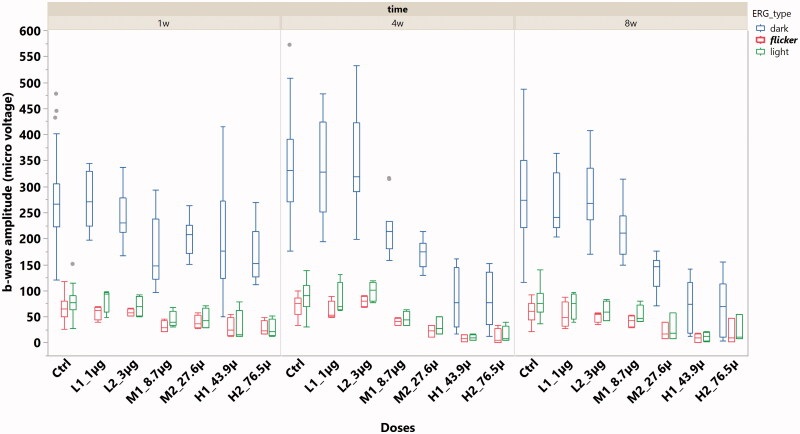
The box plot of ERG b-wave amplitude stratified by ERG types and the doses of HDP-PMEG. ctrl = control, L = low doses, M = middle doses, and H = high doses.

ERG b-wave amplitude was analyzed within the types of dark adapted, light adapted, and flicker ERGs. All three types of ERG demonstrated significant lower b-wave amplitude from the eyes with 8.7 µg or higher doses when compared with the b-wave amplitude from the control eyes or the eyes with doses lower than 8.7 µg per eye (Figure 3; *p* < .0001, GEE).

Histology examination confirmed that intravitreal injection of 3 µg HDP-PMEG was not toxic (see supplemental figure 3) In this study, inferior retina was carefully examined to reveal possible localized retina toxicity that may arise from direct touch between drug depot and retina due to gravity. Out of four eyes with 8.7 µg intravitreal dose, two eyes demonstrated the localized inferior retina toxicity although the retina elsewhere or at visual streak had normal structure under light microscopy (see supplemental figure 4). The rabbits in current ocular toxicity study were observed for 8 weeks. The mean body weight of the animals entering the study was 2.1 ± 0.17 kg and increased to 2.6 ± 0.38 kg before the sacrifice. No general toxicity was noted during the study.

### Antiproliferation in rabbit PVR model following a short period of intraocular infusion

**Figure 4.**
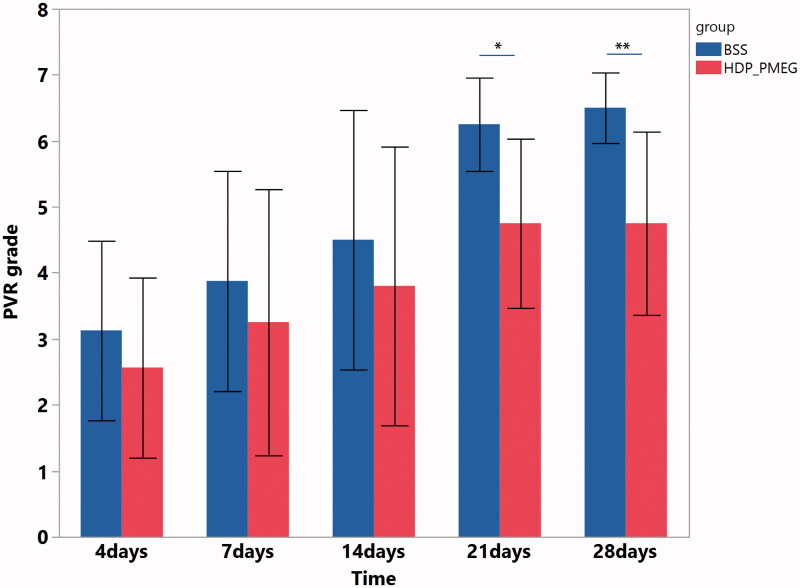
The mean PVR grading with one standard deviation from the eyes injected with HDP-PMEG (red) or BSS (blue) at the different exam time points. *: Indicate statistically significant with *p* < .05; **: Indicate statistically significant with *p* ≤ .01.

PVR was graded on day 4, day 7, day 14, day 21, and day 28. Due to the repeated measuring feature and ordinal grading scale, the pooled data were analyzed using SAS GEMOD procedure with link function of cumulative logit. The GEE revealed that the eyes infused with 50 min of HDP-PMEG had significantly lower PVR grades than that of the eyes infused with only BSS (BSS vs. HDP-PMEG: estimate = 1.14, OR = 3.1, *p* = .027) while adjusting for time effect (exam time points) that is also positively associated with the PVR grades (*β* = 0.14, *p*= <.0001). Further comparisons between the HDP-PMEG and BSS groups at each exam time point revealed that eyes infused with HDP-PMEG had significant lower mean PVR grades at day 21 (6.25 ± 0.71 for BSS vs. 4.75 ± 1.28 for HDP-PMEG, *p* = .02 Wilcoxon Rank Sums) and day 28 (6.5 ± 0.53 for BSS vs. 4.75 ± 1.39 for HDP-PMEG, *p* = .01 Wilcoxon Rank Sums) (Figure 4).

### Inhibitory effect of HDP-PMEG on laser-induced rat CNV model

**Figure 5.**
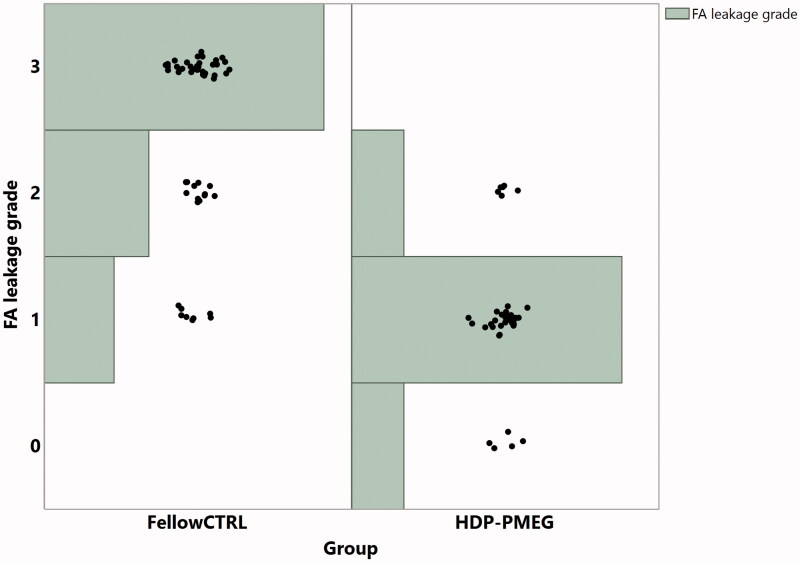
The distribution of the leakage grades of laser burns over leakage intensity and the groups, assessed at 2 weeks following the laser and intravitreal HDP-PMEG (CTRL = control; Tx = treatment with HDP-PMEG).

The laser-induced fibrous vascular proliferation was assessed on day 14 by retinal FA and was imaged on day 24 by UHR-OCT. During the study of four weeks, the mean rat weight increased from 175.5 ± 11.6 g to 252.6 ± 12.6 g, which is statistically significant (*p* < .0001, paired *t*-test). FA demonstrated that the eyes injected with HDP-PMEG had significantly less leakage than the eyes injected with BSS following laser (*β* = 0.97, OR = 38.5, *p* < .0001, GEE, Figure 5) while the extent of bleeding from the laser burns was positively associated with the grades of leakage (*β* = 1.17, *p* = .0026, GEE). Compared with the HDP-PMEG intervened eyes, the odds for BSS intervened eyes to have higher-rated FA leaking grades were 38.5 times higher.

**Figure 6.**
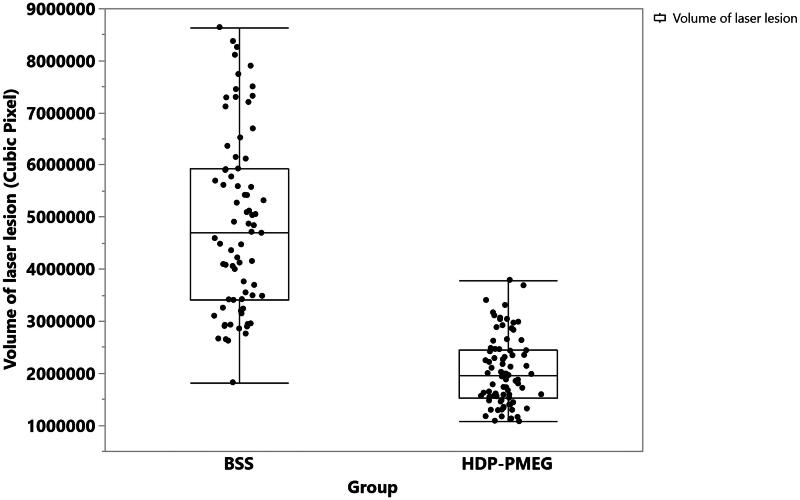
The distribution of the laser lesions volumes and the mean volumes with one standard deviation stratified by the groups.

UHR-OCT revealed that the mean volume of laser-induced fibrosis in BSS injected eyes was significantly larger than that in HDP-PMEG injected eyes (Figure 6) while the bleeding of laser burns was justified in the regression (Least Sq Mean for BSS = 4763569.5 vs. Least Sq Mean for HDP-PMEG = 2148129.7, *p* < .0001, GEE). The fibrous tissue volume of the laser burns reduced by 55% with intervention of HDP-PMEG. The bleeding of laser burns showed a trend of higher volume with more bleeding but was not statistically significant (*p* = .1).

The IOP at the sacrifice was recorded as 19.1 ± 3 for HDP-PMEG injected eyes and as 20.1 ± 4 mmHg for BSS injected eyes (*p* = .4, paired *t*-test). ERG was performed as previously reported (Dai et al., 2014) before the sacrifice 4 weeks after the laser. ERGs did not show difference between the right eyes (HDP-PMEG injected) and the left eyes (BSS injected) in any type of ERGs.

## Discussion

PVR is a blinding eye disease which is responsible for 10%–20% failure of retinal detachment repair, (Heimann et al., 2007) and PVR is also responsible for over 21% of severe vision loss after open globe eye trauma (Cardillo et al., 1997). Since the pathology of PVR cannot be reversed, identification of compounds that can prevent PVR after the primary surgical repair process has been an active area of research (Wubben et al., 2016). Among the compounds tested in clinical trials (including antimetabolites and steroids), 5-fluorouracil and daunorubicin were the only two drugs that showed some benefit of preventative efficacy (Wiedemann et al., 1998; Wubben et al., 2016). So far there is no effective treatment modality developed from those agents. A promising compound to limit formation of PVR needs to be pharmacologically potent on unwanted cells growing and transformation but minimally toxic to normal retinal cells. At the same time, it would be a huge advantage if the therapeutics can either be applied during the primary surgery or formulated into a sustained release suspension for a simple intravitreal injection.

For intraoperative application of an antiproliferative agent, the drug can be added into the irrigation solution used for trans pars plana vitrectomy. To be used in such a route, a compound needs to possess genotoxicity, a pharmacological property that persists after the drug is no longer present. 5-fluorouracil was tested in such a setting along with low molecular weight heparin in three clinical trials (Asaria et al., 2001; Charteris et al., 2004; Wickham et al., 2007). However, the pharmacological effect in these clinical trials was conflicting and questionable (Charteris et al., 2004; Wickham et al., 2007). Compared with 5-fluorouracil, HDP-PMEG demonstrated stronger genotoxicity in our in vitro study. The current in vivo study with 50 min of intravitreal infusion demonstrated significant pharmacological effect on rabbit PVR model. There is intrinsic difference between clinical perioperative evaluation of antiproliferation potency and the experimental evaluation on the current PVR model with intravitreal drug infusion. In a clinical setting, the participating proliferating cells such as dissociated RPE cells and in situ glial cells are exposed to the drug during the infusion. In contrast, the current rabbit model has the main proliferation from injected exogenous RPE cells that were injected after HDP-PMEG was washed away. HDP-PMEG may show better efficacy in a similar clinical testing as used for 5-fluorouracil clinical trials. The results of the two randomized clinical trials suggested the genotoxicity of 5-fluorouracil may not be strong enough to effectively inhibit the unwanted ocular cell proliferation with a short-time exposure. 5-Fluoruracill is also used for inhibiting aberrant proliferation after glaucoma filtering surgery; however, its genotoxicity is far weaker than that of mitomycin C. It has been suggested that the genotoxicity of PMEG diphosphate (active metabolite of HDP-PMEG) is comparable to that of mitomycin C (Otova et al., 1997).

In addition to the intraoperative use of an antiproliferative agent, intravitreal injection following ocular surgery or ocular trauma is also a convenient way to prevent PVR development. For this route of drug application, vitreous half-life becomes an important issue. Most intravitreal injectable antiproliferative compounds have short vitreous half-lives. For example, daunorubicin is another high profile drug in addition to 5-fluorouracil in prevention of PVR development (Wiedemann et al., 1985, 1998, Wickham et al., 2007). Daunorubicin has a vitreous half-life of only 5 h (Kizhakkethara et al., 1996). Two randomized controlled clinical trials using intravitreal one-time 5 µg daunorubicin or intraoperative 10 min infusion 7.5 µg/mL both did not yield significant clinical efficacy. However, once used with a sustained delivery system, daunorubicin significantly inhibited experimental PVR formation (Hou et al., 2015). Compared with daunorubicin, HDP-PMEG is minimally water-soluble and one safe dose (3 µg/eye in rabbit) of intravitreal suspension can last for at least 6 weeks. The 6-week sustained presence of HDP-PMEG in vitreous is very important and advantageous because the median time to develop PVR following retinal surgery or trauma is about 1.3-2 months (Mietz and Heimann, 1995; Cardillo et al., 1997). A simple intravitreal injection of HDP-PMEG following primary retinal detachment repair or primary ocular trauma repair may reduce PVR development and improve visual outcome. The current in vivo rat CNV model demonstrated that HDP-PMEG intravitreal injection (104 ng in rat eye is equivalent to 3 µg in rabbit eye) significantly suppressed both fluorescein leakage and fibrous scar formation. The grade 3 leakage was not seen in the eyes injected with HDP-MPEG, which suggested significant suppression of neovascularization within the laser burns. The data showed there was a positive association between leakage and the extent of bleeding associated with the burns, which is in agreement with the previous study report (El Bradey et al., 2004).

Rabbit has been a preferred ocular experimental animal due to the friendly personality and easy handling. However, the scientific use needs to be considered for the study purpose. Rabbit eye has been widely used for ocular pharmacokinetics studies of intravitreal drug administration or delivery systems due to the relatively closer vitreous volume and the low cost (Bakri et al., 2007; Nomoto et al., 2009). However, rabbit retina is substantially different from that of human due to the lack of complete retinal vascular system. For a PVR model, retinal vasculature may be very important because it directly participates in the formation of PVR. Evidence for this is that the first appearance of rabbit PVR is always on or around the medullary ray on which there is vascular distribution. Therefore, rabbit PVR model may not be an ideal animal model for drug screening studies. Indeed, 5-fluorouracil was effective on rabbit PVR but failed in clinical trials (Rubsamen et al., 1994; Charteris et al., 2004; Wickham et al., 2007). Another disadvantage of the rabbit PVR model is the exogenous cell injection instead of the native cell proliferation. In the current study, we used laser-induced rat CNV model to test the efficacy of HDP-PMEG. Rat CNV is a well-characterized model with cell proliferation to form an ellipsoidal fibrovascular mass that can be quantitated in a living animal by calculating from noninvasive OCT scans (Sulaiman et al., 2015). This ellipsoid volume measurements allow rapid, quantitative use of OCT for the assessment of CNV lesions in vivo in response to the experimental intervention and the measurement had good correlation with the standard ex vivo choroidal flatmounts and confocal microscopy (Sulaiman et al., 2015). In the current study, the fibrous tissue volume of the laser burns in HDP-PMEG injected eyes reduced by 55% when compared with the laser burns in the contralateral eyes.

In summary, HDP-PMEG was lipid derived from PMEG to enhance the drug potency and vitreous sustainability. HDP (hexadecyloxypropyl) is a long-chain, lipophilic, alkoxyalkyl moiety conjugated to PMEG via a labile phosphonoester bond. HDP-PMEG functions as a prodrug which, after cellular uptake, is metabolized to PMEG diphosphate that is highly effective antiproliferative metabolite. Alkoxyalkyl moiety (HDP) greatly enhanced PMEG to penetrate cell membrane and incorporate into the target cells compared to free PMEG. Free HDP is not cytotoxic. HDP-PMEG possesses cell genotoxicity that may be harnessed for intraoperative application to prevent PVR development. HDP-PMEG is minimally water-soluble, which can be an advantage to prepare sustained release formulation for intravitreal delivery following primary retinal surgery or primary repair of open globe trauma. This represents a novel strategy to use lipid prodrug as an intravitreal slow drug release system. In addition, due to the limited water solubility, HDP-PMEG suspension may be ideal for local application to inhibit tumor growth outside of eye arena to limit PMEG systemic exposure and avoid gastrointestinal and renal toxicity. Though no obvious systemic toxicity was noted from the current study, it is necessary for future study to quantitate HDP-PMEG as well as PMEG in systemic circulation in order to better gorge the systemic safety. More pharmacodynamics research will further characterize this compound for its clinical application against PVR or tumors.

## Supplementary Material

IDRD_Cheng_et_al_Supplemental_Content.zip

## Abbreviations

(PVR): Proliferative Vitreoretinopathy
(HDP-PMEG): Hexadecyloxypropyl 9-[(2-phosphonomethoxy)ethyl]guanine
(HDP): Hexadecyloxypropyl
(BSS): Balanced Salt Solution
(GEE): Generalized Estimating Equation
(CNV): Choroidal Neovascularization
(UHR-OCT): Ultra-High Resolution Optical Coherence Tomography
(RPE): Retinal Pigment Epithelium
(IOP): Intraocular Pressure
(AMD): Macular Degeneration
(ERG): Electroretinography
GCV: Ganciclovir
CDV: Cidofovir

## References

[CIT0001] Agrawal RN, He S, Spee C, et al. (2007). In vivo models of proliferative vitreoretinopathy. Nat Protoc 2:67–77. 17401340 10.1038/nprot.2007.4

[CIT0002] Aldern KA, Ciesla SL, Winegarden KL, Hostetler KY. (2003). Increased antiviral activity of 1-O-hexadecyloxypropyl-[2-(14)C]cidofovir in MRC-5 human lung fibroblasts is explained by unique cellular uptake and metabolism. Mol Pharmacol 63:678–81. 12606777 10.1124/mol.63.3.678

[CIT0003] Asaria RH, Charteris DG. (2006). Proliferative vitreoretinopathy: developments in pathogenesis and treatment. Compr Ophthalmol Update 7:179–85. 17007731

[CIT0004] Asaria RH, Kon CH, Bunce C, et al. (2001). Adjuvant 5-fluorouracil and heparin prevents proliferative vitreoretinopathy: Results from a randomized, double-blind, controlled clinical trial. Ophthalmology 108:1179–83. 11425671 10.1016/s0161-6420(01)00589-9

[CIT0005] Bakri SJ, Snyder MR, Reid JM, et al. (2007). Pharmacokinetics of intravitreal bevacizumab (Avastin). Ophthalmology 114:855–9. 17467524 10.1016/j.ophtha.2007.01.017

[CIT0006] Banerjee PJ, Quartilho A, Bunce C, et al. (2017). Slow-release dexamethasone in proliferative vitreoretinopathy: a prospective, randomized controlled clinical trial. Ophthalmology 124:757–67. 28237428 10.1016/j.ophtha.2017.01.021

[CIT0007] Beadle JR, Hartline C, Aldern KA, et al. (2002). Alkoxyalkyl esters of cidofovir and cyclic cidofovir exhibit multiple-log enhancement of antiviral activity against cytomegalovirus and herpesvirus replication in vitro. Antimicrob Agents Chemother 46:2381–6. 12121908 10.1128/AAC.46.8.2381-2386.2002 PMC127379

[CIT0008] Beadle JR, Valiaeva N, Yang G, et al. (2016). Synthesis and antiviral evaluation of octadecyloxyethyl benzyl 9-[(2-Phosphonomethoxy)ethyl]guanine (ODE-Bn-PMEG), a potent inhibitor of transient HPV DNA amplification. J Med Chem 59:10470–8. 27933957 10.1021/acs.jmedchem.6b00659

[CIT0009] Berkowitz BA, Lukaszew RA, Mullins CM, Penn JS. (1998). Impaired hyaloidal circulation function and uncoordinated ocular growth patterns in experimental retinopathy of prematurity. Invest Ophthalmol Vis Sci 39:391–6. 9477999

[CIT0010] Cardillo JA, Farah ME, Mitre J, et al. (2004). An intravitreal biodegradable sustained release naproxen and 5-fluorouracil system for the treatment of experimental post-traumatic proliferative vitreoretinopathy. Br J Ophthalmol 88:1201–5. 15317716 10.1136/bjo.2003.039917 PMC1772295

[CIT0011] Cardillo JA, Stout JT, Labree L, et al. (1997). Post-traumatic proliferative vitreoretinopathy. The epidemiologic profile, onset, risk factors, and visual outcome. Ophthalmology 104:1166–73. 9224471 10.1016/s0161-6420(97)30167-5

[CIT0012] Charteris DG, Aylward GW, Wong D, et al. (2004). A randomized controlled trial of combined 5-fluorouracil and low-molecular-weight heparin in management of established proliferative vitreoretinopathy. Ophthalmology 111:2240–5. 15582080 10.1016/j.ophtha.2004.05.036

[CIT0013] Chen M, Li XL, Liu JK, et al. (2015). Safety and pharmacodynamics of suprachoroidal injection of triamcinolone acetonide as a controlled ocular drug release model. J Control Release 203:109–17. 25700623 10.1016/j.jconrel.2015.02.021

[CIT0014] Cheng L, Hostetler K, Valiaeva N, et al. (2010). Intravitreal crystalline drug delivery for intraocular proliferation diseases. Invest Ophthalmol Vis Sci 51:474–81. 19696179 10.1167/iovs.09-3672 PMC2869063

[CIT0015] Cheng L, Hostetler KY, Chaidhawangul S, et al. (2002). Treatment or prevention of herpes simplex virus retinitis with intravitreally injectable crystalline 1-O-hexadecylpropanediol-3-phospho-ganciclovir. Invest Ophthalmol Vis Sci 43:515–21. 11818399

[CIT0016] Cheng L, Hostetler KY, Gardner MF, et al. (1999). Intravitreal toxicology in rabbits of two preparations of 1-O-octadecyl-sn-glycerol-3-phosphonoformate, a sustained-delivery anti-CMV drug. Invest Ophthalmol Vis Sci 40:1487–95. 10359331

[CIT0017] Cheng L, Hostetler KY, Lee J, et al. (2004). Characterization of a novel intraocular drug-delivery system using crystalline lipid antiviral prodrugs of ganciclovir and cyclic cidofovir. Invest Ophthalmol Vis Sci 45:4138–44. 15505067 10.1167/iovs.04-0064 PMC2666013

[CIT0018] Dai XF, Han JJ, Qi Y, et al. (2014). AAV-Mediated Lysophosphatidylcholine Acyltransferase 1 (Lpcat1) Gene Replacement Therapy Rescues Retinal Degeneration in rd11 Mice. Invest Ophthalmol Vis Sci 55:1724–34. 24557352 10.1167/iovs.13-13654 PMC3968931

[CIT0019] De Fendi LI, Arruda GV, Scott IU, Paula JS. (2013). Mitomycin C versus 5-fluorouracil as an adjunctive treatment for trabeculectomy: a meta-analysis of randomized clinical trials. Clin Clin Experiment Ophthalmol 41:798–806. 24308066 10.1111/ceo.12097

[CIT0020] El Bradey M, Cheng LY, Bartsch DU, et al. (2004). Preventive versus treatment effect of Ag3340, a potent matrix metalloproteinase inhibitor in a rat model of choroidal neovascularization. J Ocul Pharmacol Ther 20:217–36. 15279727 10.1089/1080768041223657 PMC1360230

[CIT0021] Gu B, Liu JK, Li XL, et al. (2015). Real-time monitoring of suprachoroidal space (SCS) following SCS injection using ultra-high resolution optical coherence tomography in guinea pig eyes. Invest Ophthalmol Vis Sci 56:3623–34. 26047049 10.1167/iovs.15-16597

[CIT0022] Heimann H, Bartz-Schmidt KU, Bornfeld N, et al. (2007). Scleral buckling versus primary vitrectomy in rhegmatogenous retinal detachment: a prospective randomized multicenter clinical study. Ophthalmology 114:2142–54. 18054633 10.1016/j.ophtha.2007.09.013

[CIT0023] Hida T, Chandler DB, Sheta SM. (1987). Classification of the stages of proliferative vitreoretinopathy in a refined experimental model in the rabbit eye. Graefe's Arch Clin Exp Ophthalmol 225:303–7. 3653728 10.1007/BF02150154

[CIT0024] Hou HY, Huffman K, Rios S, et al. (2015). A novel approach of daunorubicin application on formation of proliferative retinopathy using a porous silicon controlled delivery system: pharmacodynamics. Invest Ophthalmol Vis Sci 56:2755–63. 25829415 10.1167/iovs.15-16526 PMC4416660

[CIT0025] Jarus G, Blumenkranz M, Hernandez E, Sossi N. (1985). Clearance of intravitreal fluorouracil. Normal and aphakic vitrectomized eyes. Ophthalmology 92:91–6. 3974999 10.1016/s0161-6420(85)34063-0

[CIT0026] Jiang H, Abukhalil F, Shen MX, et al. (2012). Slit-lamp-adapted ultra-high resolution OCT for imaging the posterior segment of the eye. Ophthalmic Surg Lasers Imaging 43:76–81. 22251848 10.3928/15428877-20111129-03

[CIT0027] Kizhakkethara I, Li X, Elsayed S, et al. (1996). Noninvasive monitoring of intraocular pharmacokinetics of daunorubicin using fluorophotometry. Int Ophthalmol 19:363–7. 10.1007/BF00130856 8970871

[CIT0028] Klein R, Knudtson MD, Lee KE, et al. (2008). The Wisconsin epidemiologic study of diabetic retinopathy: XXII the twenty-five-year progression of retinopathy in persons with type 1 diabetes. Ophthalmology 115:1859–68. 19068374 10.1016/j.ophtha.2008.08.023 PMC2761813

[CIT0029] Koh HJ, Bessho K, Cheng LY, et al. (2004). Inhibition of choroidal neovascularization in rats by the urokinase-derived peptide A6. Invest Ophthalmol Vis Sci 45:635–40. 14744908 10.1167/iovs.03-0735 PMC1378117

[CIT0030] Kramata P, Downey KM, Paborsky LR. (1998). Incorporation and excision of 9-(2-phosphonylmethoxyethyl)guanine (PMEG) by DNA polymerase delta and epsilon *in vitro*. J Biol Chem 273:21966–71. 9705337 10.1074/jbc.273.34.21966

[CIT0031] Krzystolik MG, Afshari MA, Adamis AP, et al. (2002). Prevention of experimental choroidal neovascularization with intravitreal anti-vascular endothelial growth factor antibody fragment. Arch Ophthalmol 120:338–46. 11879138 10.1001/archopht.120.3.338

[CIT0032] Kwak HW, D’amico DJ. (1992). Evaluation of the retinal toxicity and pharmacokinetics of dexamethasone after intravitreal injection. Arch Ophthalmol 110:259–66. 1736876 10.1001/archopht.1992.01080140115038

[CIT0033] Mietz H, Heimann K. (1995). Onset and recurrence of proliferative vitreoretinopathy in various vitreoretinal diseases. British Journal of Ophthalmology 79:874–7. 7488572 10.1136/bjo.79.10.874 PMC505285

[CIT0034] Nomoto H, Shiraga F, Kuno N, et al. (2009). Pharmacokinetics of bevacizumab after topical, subconjunctival, and intravitreal administration in rabbits. Invest Ophthalmol Vis Sci 50:4807–13. 19324856 10.1167/iovs.08-3148

[CIT0035] Otova B, Holy A, Votruba I, et al. (1997). Genotoxicity of purine acyclic nucleotide analogs. Folia Biol (Praha) 43:225–9. 9595265

[CIT0036] Otova B, Hrdy J, Votruba I, Holy A. (2009). *In vivo* modulation of angiogenic gene expression by acyclic nucleoside phosphonates PMEDAP and PMEG. Anticancer Res 29:1295–302. 19414378

[CIT0037] Rahimy MH, Peyman GA, Fernandes ML, et al. (1994). Effects of an intravitreal daunomycin implant on experimental proliferative vitreoretinopathy: simultaneous pharmacokinetic and pharmacodynamic evaluations. J Ocul Pharmacol 10:561–70. 7836865 10.1089/jop.1994.10.561

[CIT0038] Reiser H, Wang J, Chong L, et al. (2008). GS-9219–a novel acyclic nucleotide analogue with potent antineoplastic activity in dogs with spontaneous non-Hodgkin’s lymphoma. Clin Cancer Res 14:2824–32. 18451250 10.1158/1078-0432.CCR-07-2061

[CIT0039] Rubsamen PE, Davis PA, Hernandez E, et al. (1994). Prevention of experimental proliferative vitreoretinopathy with a biodegradable intravitreal implant for the sustained release of fluorouracil. Arch Ophthalmol 112:407–13. 8129669 10.1001/archopht.1994.01090150137036

[CIT0040] Sha O, Kwong WH. (2006-07). Postnatal developmental changes of vitreous and lens volumes in sprague-Dawley Rats. Neuroembryol Aging 4:183–8.

[CIT0041] Stern WH, Guerin CJ, Erickson PA, et al. (1983). Ocular toxicity of fluorouracil after vitrectomy. Am J Ophthalmol 96:43–51. 6869479 10.1016/0002-9394(83)90453-1

[CIT0042] Sulaiman RS, Quigley J, Qi XP, et al. (2015). A simple optical coherence tomography quantification method for choroidal neovascularization. J Ocul Pharmacol Ther 31:447–54. 26060878 10.1089/jop.2015.0049 PMC4598937

[CIT0043] Thamm DH, Vail DM, Kurzman ID, et al. (2014). GS-9219/VDC-1101-a prodrug of the acyclic nucleotide PMEG has antitumor activity in spontaneous canine multiple myeloma. Bmc Vet Res 10:30. 24460928 10.1186/1746-6148-10-30 PMC3904015

[CIT0044] Valiaeva N, Beadle JR, Aldern KA, et al. (2006). Synthesis and antiviral evaluation of alkoxyalkyl esters of acyclic purine and pyrimidine nucleoside phosphonates against HIV-1 in vitro. Antiviral Res 72:10–9. 16630664 10.1016/j.antiviral.2006.03.007

[CIT0045] Wang FH, Liang YB, Zhang F, et al. (2009). Prevalence of diabetic retinopathy in rural China: the Handan Eye Study. Ophthalmology 116:461–7. 19168222 10.1016/j.ophtha.2008.10.003

[CIT0046] Wickham L, Bunce C, Wong D, et al. (2007). Randomized controlled trial of combined 5-Fluorouracil and low-molecular-weight heparin in the management of unselected rhegmatogenous retinal detachments undergoing primary vitrectomy. Ophthalmology 114:698–704. 17398320 10.1016/j.ophtha.2006.08.042

[CIT0047] Wiedemann P, Hilgers RD, Bauer P, Heimann K. (1998). Adjunctive daunorubicin in the treatment of proliferative vitreoretinopathy: results of a multicenter clinical trial. Daunomycin Study Group. Am J Ophthalmol 126:550–9. 9780100 10.1016/s0002-9394(98)00115-9

[CIT0048] Wiedemann P, Sorgente N, Bekhor C, et al. (1985). Daunomycin in the treatment of experimental proliferative vitreoretinopathy. Effective doses in vitro and in vivo. Invest Ophthalmol Vis Sci 26:719–25. 3997421

[CIT0049] Wubben TJ, Besirli CG, Zacks DN. (2016). Pharmacotherapies for retinal detachment. Ophthalmology 123:1553–62. 27040150 10.1016/j.ophtha.2016.02.040

[CIT0050] Zhu DX, Shen MX, Jiang H, et al. (2011). Broadband superluminescent diode-based ultrahigh resolution optical coherence tomography for ophthalmic imaging. J Biomed Optics 16:1206006. 10.1117/1.3660314 PMC3247935 22191923

