# Supplementary figures and images for: A novel lipid prodrug strategy for sustained delivery of hexadecyloxypropyl 9-[2-(phosphonomethoxy)ethyl]guanine (HDP-PMEG) on unwanted ocular proliferation
Source: Drug Deliv. 2017 Nov 8;24(1):1703–12. doi: 10.1080/10717544.2017.1399303 (PMC8241053; doi:10.1080/10717544.2017.1399303)

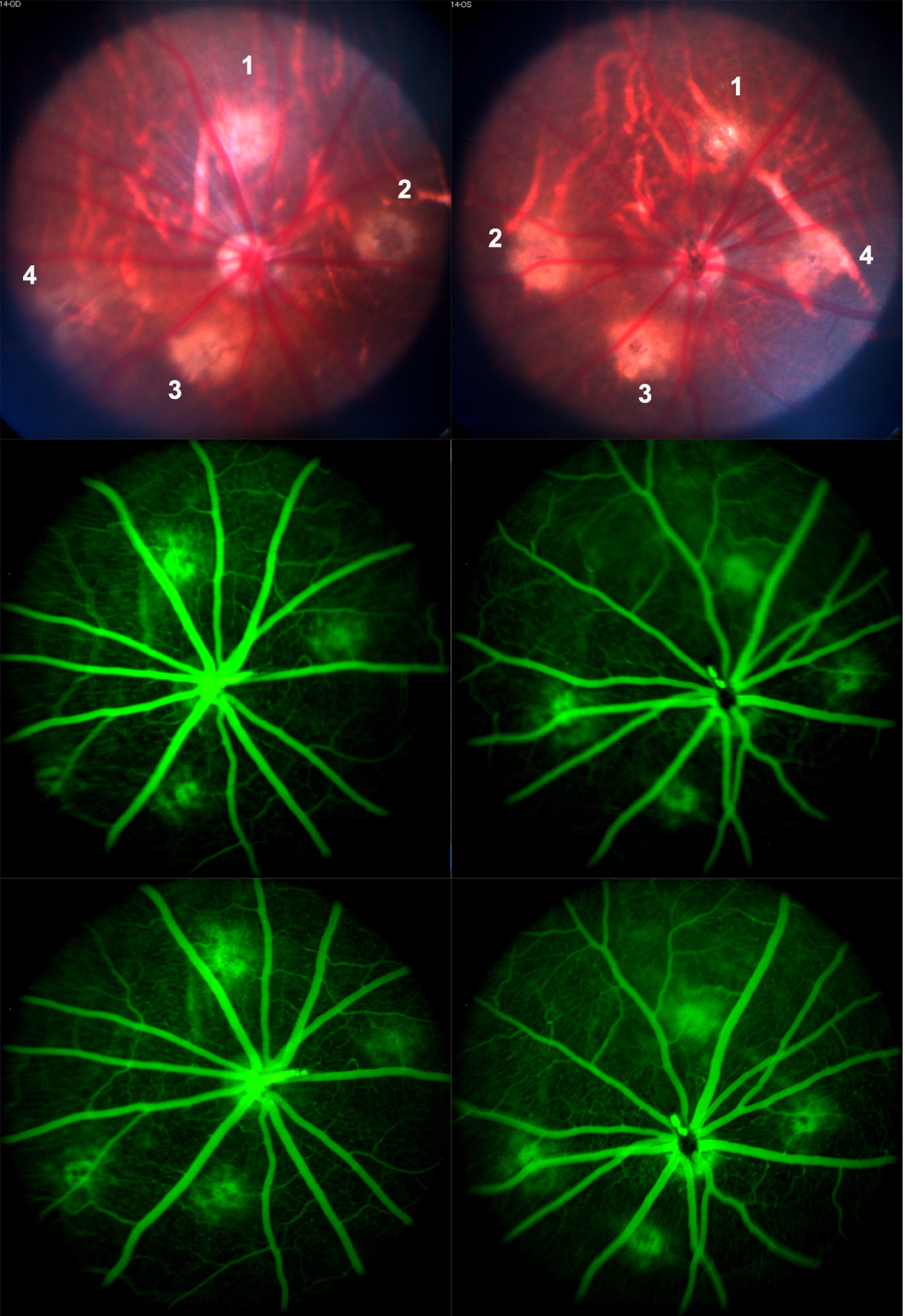

Supplement: IDRD_Cheng_et_al_Supplemental_Content.zip [file IDRD_A_1399303_SM2320.zip › Supplemental Figure 1 copy.tif]

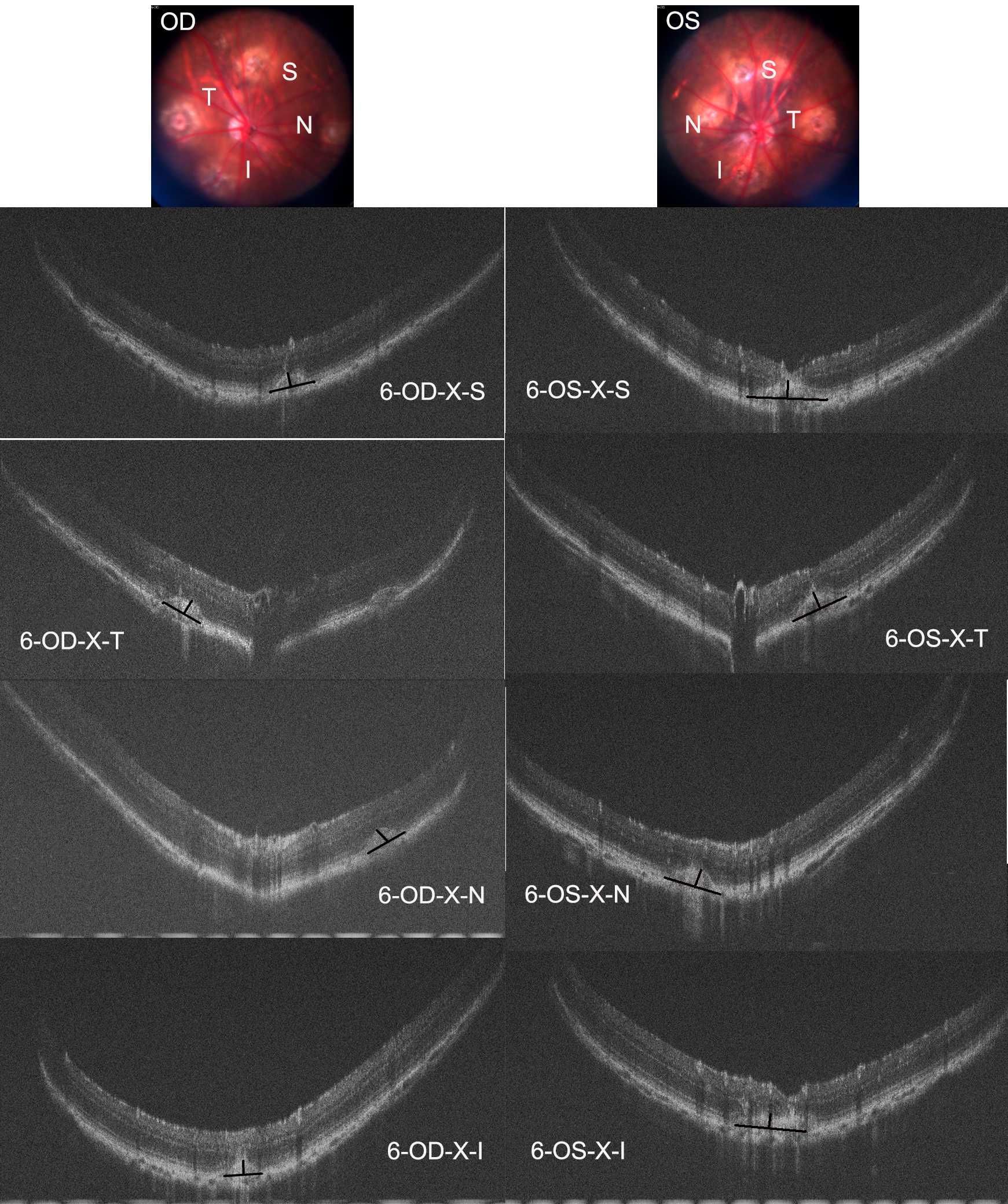

Supplement: IDRD_Cheng_et_al_Supplemental_Content.zip [file IDRD_A_1399303_SM2320.zip › Supplemental Figure 2 copy.tif]

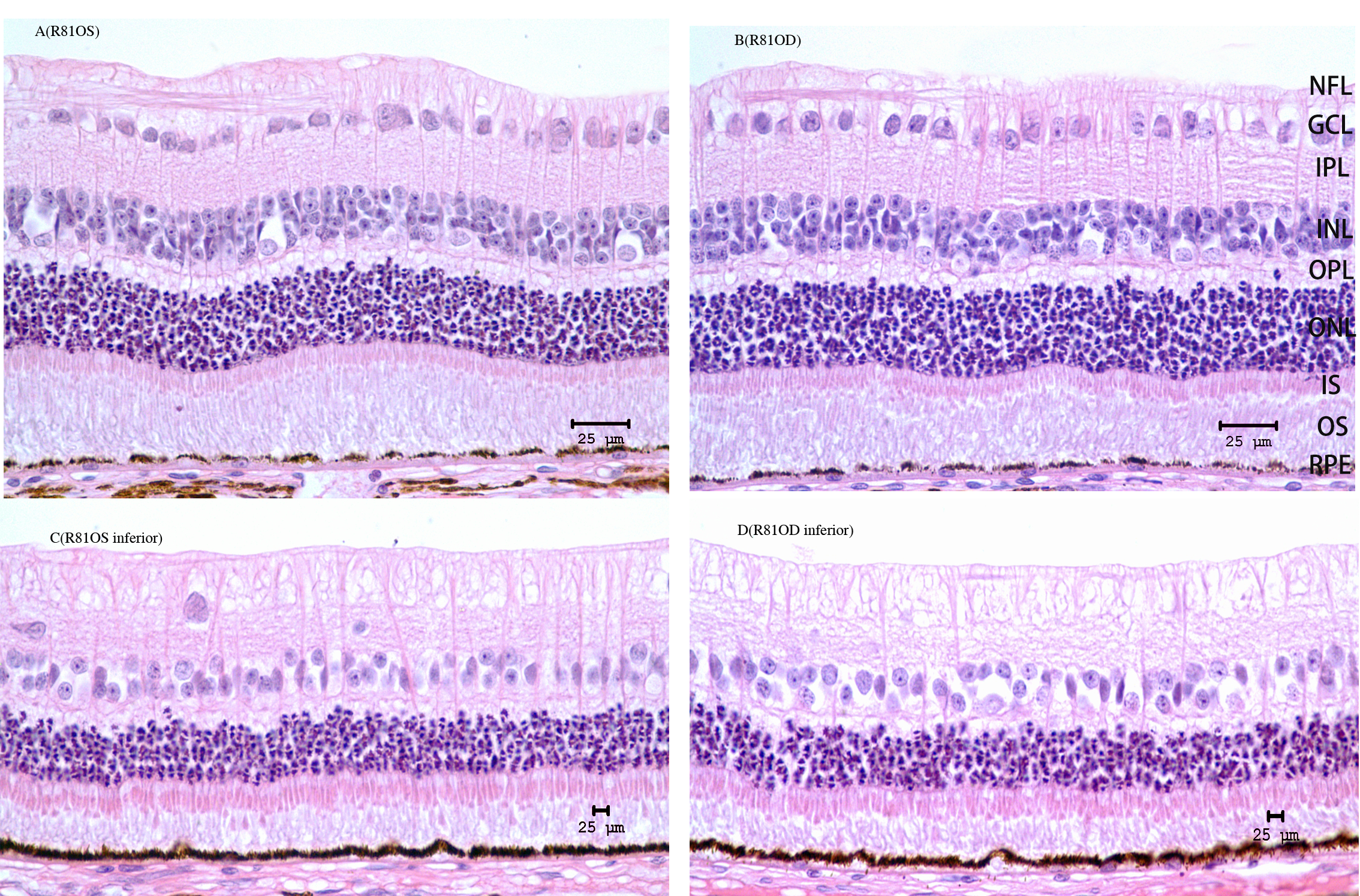

Supplement: IDRD_Cheng_et_al_Supplemental_Content.zip [file IDRD_A_1399303_SM2320.zip › Supplemental Figure 3 copy.tif]

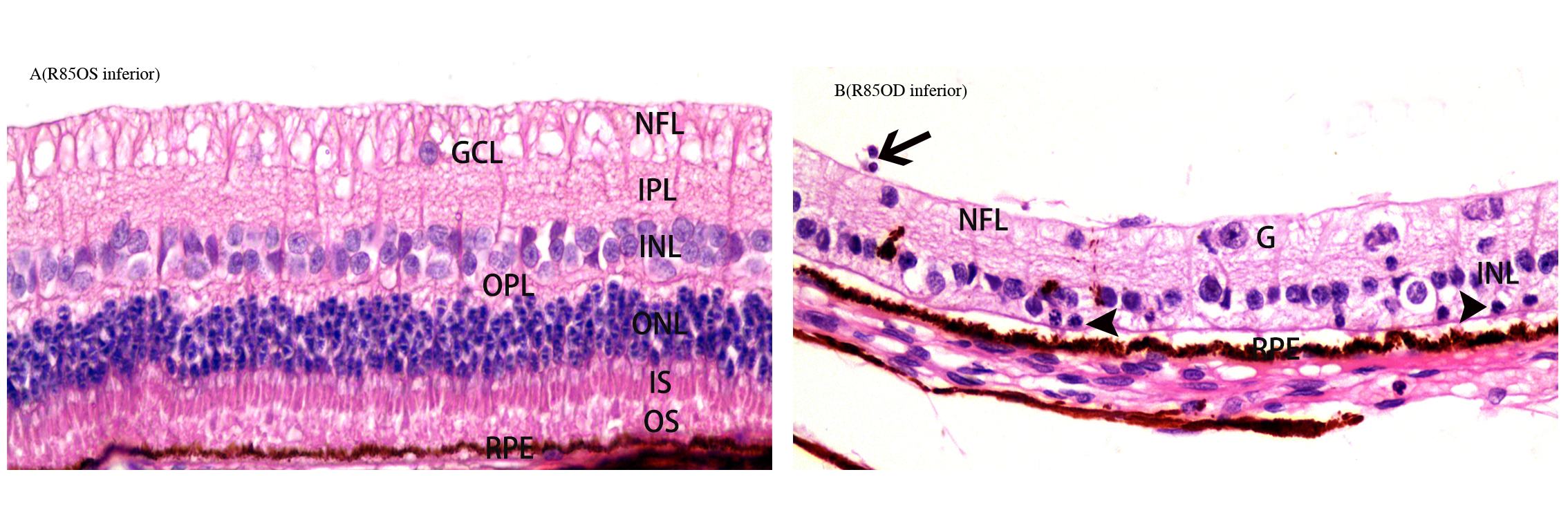

Supplement: IDRD_Cheng_et_al_Supplemental_Content.zip [file IDRD_A_1399303_SM2320.zip › Supplemental Figure 4 copy.tif]
